# Early detection of treatment futility in patients with metastatic colorectal cancer

**DOI:** 10.18632/oncotarget.28165

**Published:** 2022-01-07

**Authors:** Jodi I. Rattner, Karen A. Kopciuk, Hans J. Vogel, Patricia A. Tang, Jeremy D. Shapiro, Dongsheng Tu, Derek J. Jonker, Lillian L. Siu, Chris J. O’Callaghan, Oliver F. Bathe

**Affiliations:** ^1^Arnie Charbonneau Cancer Institute, Cumming School of Medicine, University of Calgary, Calgary, Canada; ^2^Department of Mathematics and Statistics, Faculty of Science, University of Calgary, Calgary, Canada; ^3^Department Biological Sciences, Faculty of Science, University of Calgary, Calgary, Canada; ^4^Department of Surgery and Oncology, Cumming School of Medicine, University of Calgary, Calgary, Canada; ^5^Department of Medical Oncology, Monash University, Melbourne, Victoria, Australia; ^6^Department of Community Health and Epidemiology, Queens University, Kingston, Canada; ^7^Division of Medical Oncology, Ottawa Hospital Cancer Centre, Ottawa, Canada; ^8^Division of Medical Oncology and Hematology, Princess Margaret Cancer Centre, Toronto, Canada

**Keywords:** colorectal cancer, metabolomics, response biomarkers, radiographic imaging, chemotherapy

## Abstract

Purpose: Chemotherapy options for treating CRC have rapidly expanded in recent years, and few have predictive biomarkers. Oncologists are challenged with evidence-based selection of treatments, and response is evaluated retrospectively based on serial imaging beginning after 2–3 months. As a result, cumulative toxicities may appear in patients who will not benefit. Early recognition of non-benefit would reduce cumulative toxicities. Our objective was to determine treatment-related changes in the circulating metabolome corresponding to treatment futility.

Methods: Metabolomic studies were performed on serial plasma samples from patients with CRC in a randomized controlled trial of cetuximab vs. cetuximab + brivanib (*N* = 188). GC-MS quantified named 94 metabolites and concentrations were evaluated at baseline, Weeks 1, 4 and 12 after treatment initiation. In a discovery cohort (*N* = 68), a model distinguishing changes in metabolites associated with radiographic disease progression and response was generated using OPLS-DA. A cohort of 120 patients was used for validation of the model.

Results: By one week after treatment, a stable model of 21 metabolites could distinguish between progression and partial response (R2Y = 0.859; Q2Y = 0.605; *P* = 5e-4). In the validation cohort, patients with the biomarker had a significantly shorter OS (*P* < 0.0001). In a separate cohort of patients with HCC on axitinib, appearance of the biomarker also signified a shorter PFS (1.7 months vs. 9.2 months, *P* = 0.001).

Conclusion: We have identified changes in the metabolome that appear within 1 week of starting treatment associated with treatment futility. The novel approach described is applicable to future efforts in developing a biomarker for early assessment of treatment efficacy.

## INTRODUCTION

Colorectal cancer (CRC) is the second most common cause of cancer death worldwide [1]. Chemotherapy is the therapeutic mainstay in the setting of metastatic disease, present in about 25% of CRC patients. Drug choices are numerous. First-line therapy is mostly comprised of combinations of cytotoxic agents [2]. Molecular targeted agents including bevacizumab, cetuximab and panitumumab are now in common use [3, 4]. Regorafenib and trifluridine tipiracil also appear to benefit some [5–8], and niche drugs for cancers with specific molecular features are also becoming available. Currently, oncologists select treatments empirically with high levels of uncertainty of clinical benefit, and the likelihood of benefit decreases with each successive line of systemic therapy. In addition to a falling likelihood of response with later lines of therapy, toxicities accrued because of earlier lines of chemotherapy cause a decline in general health and deterioration in quality of life, causing a cumulative reduction in the proportion of patients who will be fit to receive newer agents. Therefore, if one could limit exposure to unbeneficial drugs in the first lines of therapy, it is conceivable that toxicities would have less impact on the health of each patient, allowing more opportunities to try other drugs, perhaps also increasing the odds of benefit with these newer agents.

Typically, in clinical practice, benefit from chemotherapy is estimated by performing cross-sectional imaging 2–3 months after treatment initiation. By that time, significant toxicities may have appeared. Repeated radiological imaging is expensive, and there are limitations to CT and MRI in gauging response. Size-based criteria such as RECIST [7, 8] underestimate response when targeted agents are used. Evidence of tumor progression also appears in a delayed fashion, often after clinical deterioration. Therefore, there is a need for a simple, inexpensive, convenient test that provides relatively immediate information on the biological activity (or lack of activity) of a treatment.

Treatment response is known to have metabolic consequences. For example, chemotherapy can result in diminished FDG uptake in tumors, reflecting a reduction in glycolysis [9–14]. Harnessing that principle, recently we reported on changes in the circulating metabolome that accompanied a response to chemotherapy agents [15]. However, therapeutic decisions hinge more on identifying whether a treatment is futile than on whether there is a measurable response. Early detection of treatment futility would serve to reduce exposure to potentially toxic drugs, preserving health and quality of life so that alternative treatments could be explored.

Our objective was to identify treatment-related metabolomic changes that preceded radiographic evidence of tumor progression. To accomplish this, we acquired serial blood samples from patients with metastatic CRC who participated in a randomized trial of two third-line targeted systemic therapies: cetuximab vs. cetuximab and brivanib [16].

## RESULTS

### Metabolite changes related to tumor progression appear one week after treatment initiation

**Figure 1:**
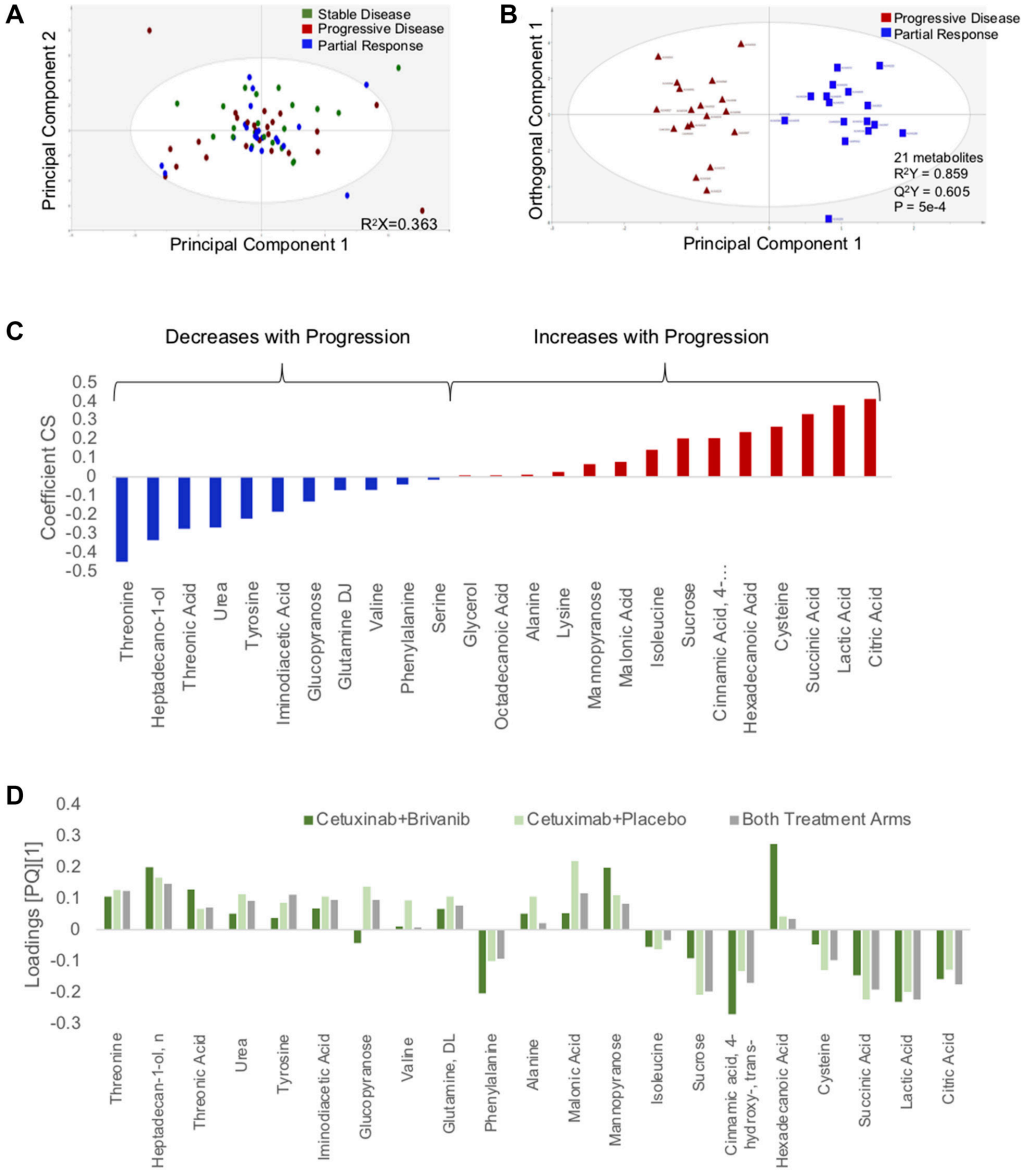
Changes in circulating metabolites are identifiable 1 week after initiation of chemotherapy. (**A**) PCA scatter plot depicting changes in plasma metabolites as a function of treatment response. (**B**) Supervised (OPLS-DA) scores scatter plot based on a model distinguishing changes in the circulating metabolome that accompany disease progression and response to chemotherapy. (**C**) Coefficient column plot describing directionality of changes of individual metabolites in association with disease progression on chemotherapy. (**D**) Loadings column plot depicting behavior of individual metabolites in each treatment arm.

The discovery cohort consisted of 68 patients, summarized in Table 1. GC-MS detected 386 compounds in all samples; of those, 94 comprised named metabolites. The PCA of the discovery set demonstrated 5 outliers (Figure 1A), which were excluded from further analysis. Metabolite changes associated with radiographic progression were compared to changes associated with measurable treatment response to identify an array of metabolite changes that signified treatment futility.

**Table 1:** Clinical factors of patients in discovery and validation cohort

Clinical Factors	Discovery Cohort (*N* = 68)	Validation Cohort (*N* = 120)
Age (Years)	60.6 ± 6.8	63.7 ± 5.6
Gender		
Male	36 (53%)	80 (66.7%)
Female	32 (47%)	37 (30.8%)
Unknown		3 (2.5%)
Response Category		
PR	22 (32.3%)	6 (5%)
PD	24 (35.3%)	33 (27.5%)
SD	22 (32.3%)	73 (60.8%)
Treatment Arm		
Cetuximab+Placebo	35 (51.5%)	59 (49.2%)
Cetuximab+Brivanib	33 (48.5%)	61 (50.8%)

A stable model that distinguished progression and response could not be derived when patients from only one treatment arm were analyzed. This may have been due to insufficient power. PCA analysis showed no distinguishable clusters related to the treatment arm (Supplementary Figure 1). Therefore, samples from both treatment arms were analyzed as a single group. PCA also did not demonstrate an intrinsic pattern related to treatment response (Figure 1A). After filtering by VIP>1 and *p*-value <0.05, a parsimonious model consisting of 21 metabolites was generated: R^2^Y score = 0.859, Q^2^Y score = 0.605, CV-ANOVA = 5 × 10^−4^ (Figure 1B). The directionality of changes in metabolites that distinguished progression and response is summarized in the coefficient plot (Figure 1C). Age and sex did not have an influence on these changes.

To confirm that the progression-related changes in the circulating metabolome applied to both treatment arms, the model was applied separately to patients from each treatment arm. The loadings plots from the whole group were similar in structure to the loadings plots of each treatment group (Figure 1D). That is, each metabolite in the progression biomarker behaved in a similar fashion in each treatment arm. Based on these findings, it appears that the progression biomarker is agnostic to treatment arm.

We took a similar approach to samples acquired at Week 4 and Week 12 after treatment initiation (Supplementary Figure 2). In these later time periods, changes in the plasma metabolome in comparison to baseline became much more variable. As a result, we were unable to generate a stable and satisfactory model that distinguished PD and PR. This is to some extent expected, as these patients have quite divergent courses that may include variable toxicities and treatment dose modifications.

### Serial monitoring for progression biomarker

The S-score (range 0–1) is a construct in the SIMCA-P software that reflects the similarity of the metabolomic profile of an individual sample to the model generated using Week 1 samples (or, in the case of our study, the metabolomic changes from baseline). S-scores in patients with radiographic PD and PR are summarized in Supplementary Figure 2. On Week 1, S-scores associated with progression ranged from 0.7–1.0; S-scores associated with treatment response ranged from 0.0–0.66. In 19 of the PR cases (86.3%), S-scores were <0.4; in the other three cases, S-scores dropped in the subsequent time period (Week 4) to <0.4. Based on these observations, we generated definitions of metabolomic biomarkers of progression and response that would be applied to the validation set. PD was defined as 2 consecutive S-scores >0.7. PR was defined as 2 or more scores <0.4.

**Figure 2:**
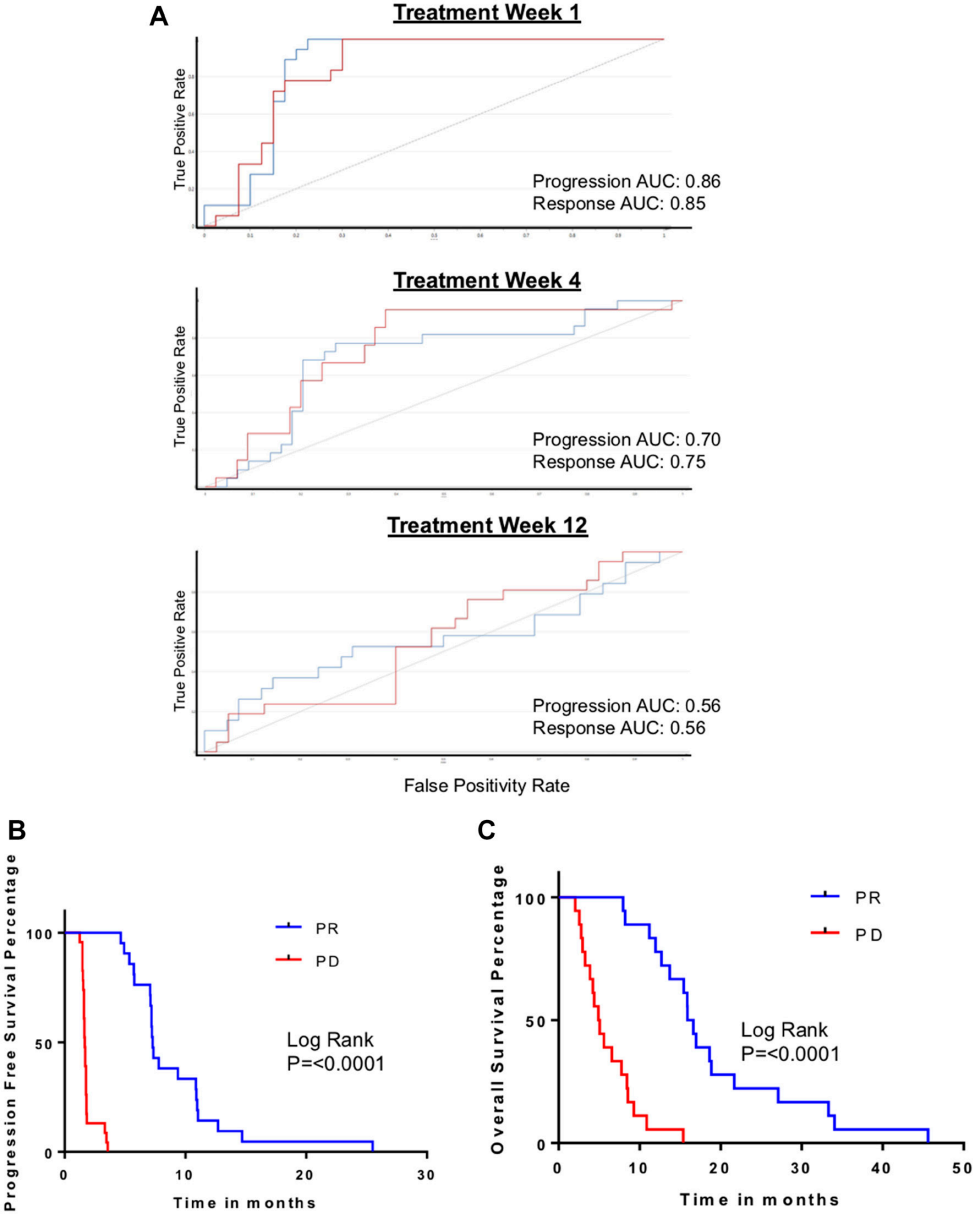
Biomarker-defined progression vs. response, based on S-scores. (**A**) ROC curves for validation of metabolomic biomarker of progression performance serially at post treatment week 1, 4 and 12 in independent validation cohort. (**B**) Kaplan–Meyer curves comparing progression-free survival (PFS) in patients with the metabolomic biomarkers of progression (PD) and response to treatment (PR). (**C**) Kaplan–Meyer curves comparing overall survival (OS) in patients with the metabolomic biomarkers of PD and PR.

The S-score values were dynamic and changed over time in each case. In 3 patients who initially had metabolomic changes associated with treatment response, the progression biomarker appeared in week 12. We considered that this reflected the emergence of resistant disease. However, given the deterioration in performance of the biomarkers past the Week 1 time point (Figure 2A), we could not confidently use the biomarker to track patients serially over time. We also explored applying a running baseline, to see if the progression biomarker appeared between intervals. Again, results were spurious and no conclusions could be made. In all, we could not confidently identify metabolomic changes that convincingly signified the emergence of chemoresistance in this cohort.

To further assess the biomarkers we defined, we evaluated their association with PFS and OS. PFS and OS were significantly and markedly different in patients who had biomarker-defined progression in comparison to patients with biomarker-defined response (Figure 2B, 2C). Specifically, PFS was 10.6 months when the PR biomarker was present and only 1.6 months when the PD biomarker appeared (*p* < 0.0001). OS was 14.3 months if the PR biomarker appeared and 3.9 months when the PD biomarker was present (*p* = 0.0002).

### Subclassification of stable disease patients

In the CO.20 trial, 47% of patients did not have radiographic criteria of either a response from chemotherapy or disease progression; they were classified as having stable disease. What remains unclear is whether their stable disease was a result of indolent tumor biology or due to benefit from chemotherapy. We postulated that patients who received benefit from the chemotherapy would have changes in the circulating metabolome that were more similar to the PR biomarker. Conversely, SD patients with indolent tumor biology would have treatment-related changes in the metabolome more similar to PD.

**Figure 3:**
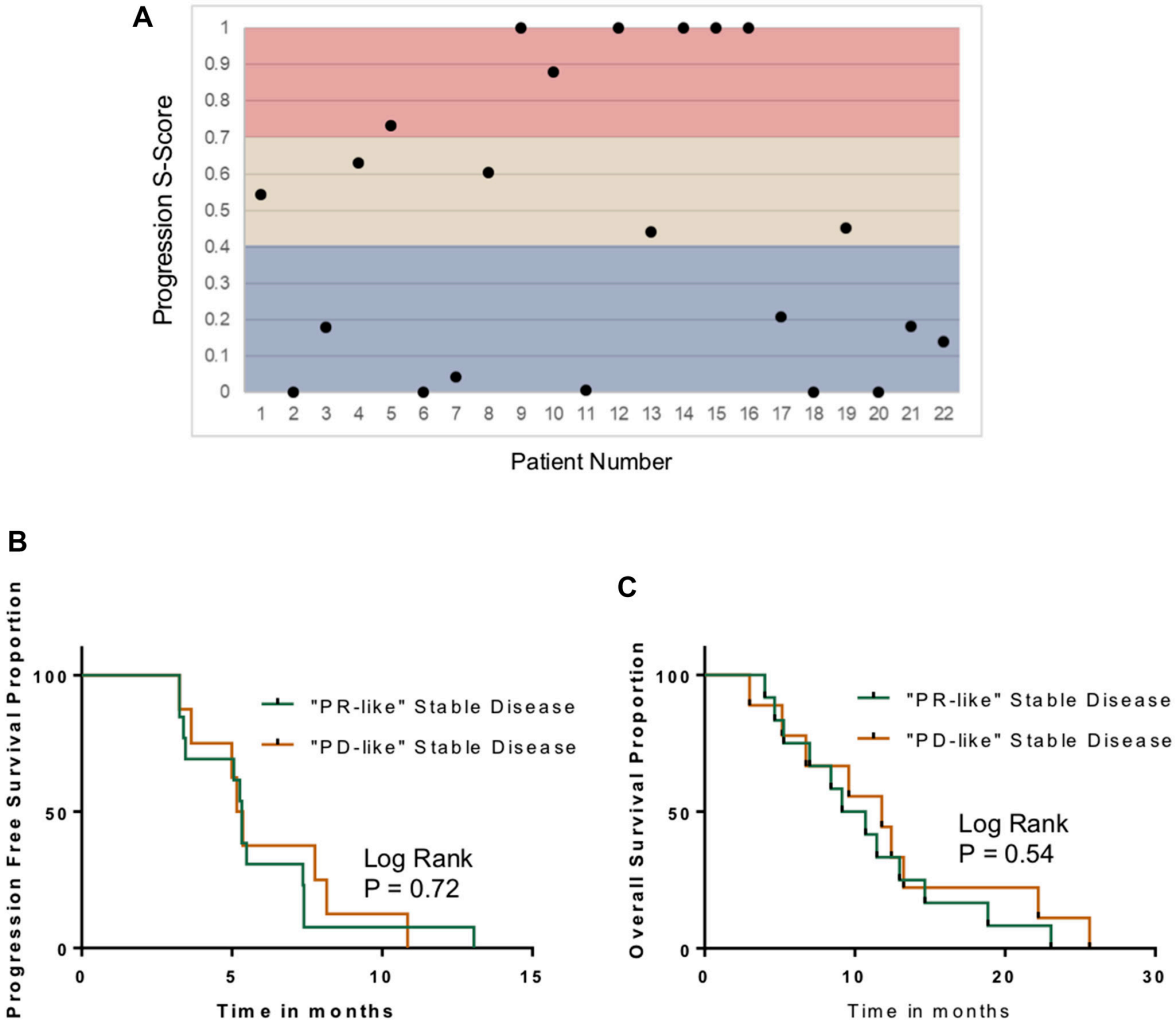
Characterization of stable disease. (**A**) Distribution of S-scores based on Week #1 changes in the plasma metabolomic in the stable disease cohort. (**B**) Kaplan–Meyer curves comparing PFS in patients with “PD-like” SD and “PR-like” stable disease, based on the S-Score system. (**C**) Kaplan–Meyer curves comparing OS in patients with “PD-like” SD and “PR-like” stable disease.

The metabolomic model for disease progression was applied to the 22 patients with radiographic stable disease. Figure 3A depicts their S-score distribution. In the discovery cohort, 5 of 22 patients with radiographic stable disease (23%) had S-scores that were in the intermediate range (i.e., neither progression nor response; 0.4<S-score<0.7). We then dichotomized the group to cases that were more similar to progression (“PD-like”) and cases that more closely resembled a treatment response (“PR-like”). To explore whether this was an effective way of classifying stable disease patients, we compared survivals. Neither PFS nor OS were significantly different in the two subgroups (Figure 3B, 3C).

### Biomarker validation

**Figure 4:**
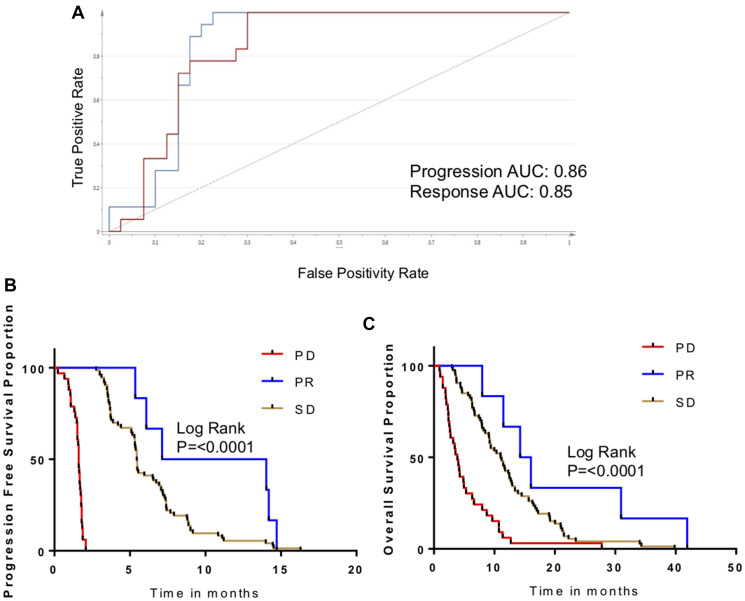
Validation of metabolomic biomarker for disease progression. (**A**) ROC curve measuring performance of metabolomic biomarker for PD/PR in the validation cohort. (**B**) Kaplan-Meier curves for patients in validation cohort, comparing PFS as a function of biomarker response classification. (**C**) Kaplan-Meier curves for patients in validation cohort, comparing OS as a function of biomarker response classification.

The metabolomic biomarkers for progression and response were subjected to validation in an independent cohort from the same clinical trial (*N* = 120). First, metabolomic biomarkers were compared to radiographic response. Overall, the progression signature had a sensitivity of 85% and a specificity of 86% in Week 1 samples (Figure 4A). As in the discovery cohort, sensitivity and specificity declined in samples acquired during later time points.

Survivals were evaluated as a function of metabolomic biomarkers (Figure 4B, 4C). As seen in the discovery cohort, PFS was markedly and significantly inferior in patients with the PD metabolomic biomarker in comparison to patients who had the PR biomarker (6.5 months vs. 1.8 months; *p* < 0.0002). Patients with an intermediate S-score (designated SD) also had an intermediate PFS. The OS followed similar trends (*P* < 0.0001).

### Progression-related changes in metabolism

The treatment-related changes in metabolites that distinguished tumor progression from chemoresponse were synthesized to devise an understanding of the metabolic processes that drove our biomarker. Tumor progression was associated with a reduction in glycolysis precursor glucopyranose and increases in lactic acid, reflecting accelerated glycolysis and the Warburg effect. Progression is also associated with a reduction in circulating glutamine, a preferred fuel for cancer cells, possibly reflecting its consumption. A general increase in TCA cycle intermediates, including succinic acid and citric acid, may also reflect increased energy demands. Decreased levels of aromatic amino acids (tyrosine and phenylalanine) were observed. In contrast, branched chain amino acids (BCAAs) were increased. Elevated circulating BCAAs have previously been reported in a number of tumor types [17]; this may be a product of skeletal muscle wasting. In addition, the capability of the tumor to catabolize BCAAs varies widely, and impaired BCAA breakdown may be a contributing factor [17]. Finally, urea levels are decreased in association with progression, which may reflect the dysregulation of the urea cycle. Previous studies have shown that urea cycle dysregulation occurs across a broad array of cancer types and supports cancer cell proliferation [18]. In all, the changes in the circulating metabolome that accompany disease progression reflect processes that support the metabolic requirements of proliferating cancer cells.

### Potential relevance in other tumor types

**Figure 5:**
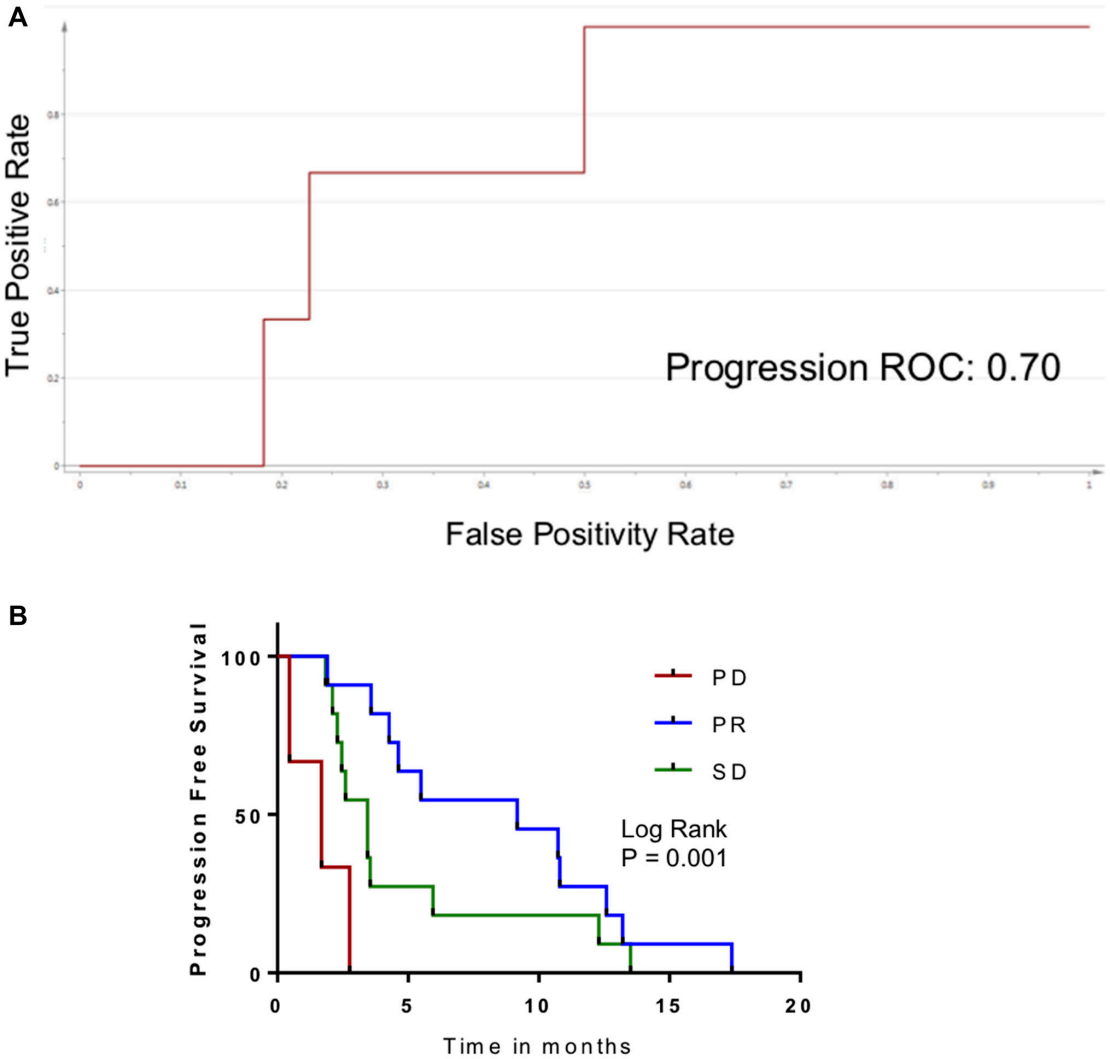
Application of metabolomic biomarker for disease progression in patients treated with axitinib. (**A**) ROC curve measuring performance of metabolomic biomarker for PD in HPCC patients. (**B**) Kaplan–Meier curve for patients in external cohort, comparing PFS as a function of biomarker response classification.

Because the progression biomarker contained features that had biological relevance to tumor growth in general and because it was applicable to patients who had two different systemic therapies, we wanted to explore its generalizability. We had previously reported on a series of 24 patients with hepatocellular carcinoma treated with second-line axitinib [15]. Serial samples consisted of serum (as opposed to plasma, as in the current series). Metabolomic criteria for progression were present in 4 patients, and metabolomic criteria for response were present in 9 patients. When these biomarkers were evaluated as a function of radiographic response, AUROC was 0.70 for the progression biomarker. However, there were only 3 patients who had progressive disease in that series. The AUROC for the metabolomic biomarker of response was 0.79 (Figure 5A). The relationship of these biomarkers to PFS was significant. Median PFS was only 1.7 months in patients who had the metabolomic changes associated with progression, and 9.2 months for patients who had criteria of response (*P* = 0.001; Figure 5B).

## DISCUSSION

As therapeutic options for cancer expand, oncologists are challenged with selecting the best treatment for each individual patient. Ideally, predictive biomarkers that identify individuals who will or will not respond to a given drug will aid in the selection. However, for the vast majority of drugs, such biomarkers are unavailable. Therefore, a common clinical approach is to select a drug or drug combination based on best evidence, then administer the drug until either dose-limiting toxicities appear or until there is radiographic evidence of disease progression. This approach may adversely impair quality of life while administering an unbeneficial treatment. Additionally, the payer is strapped with the costs of drug(s), treatment of toxicities, and the costs of serial CT scans or MRI scans. An alternative approach is to devise a method to identify patients who will not benefit from a treatment as soon as possible after treatment initiation, before substantial toxicities and costs are incurred.

To this end, we describe a biomarker-based approach for early identification of treatment futility that involves serial monitoring of the circulating metabolome. Within a week of initiating systemic therapy, features of tumor progression appear. In some instances, those features do not appear until 4 weeks after treatment initiation; repeated appearance of those metabolomic features is highly predictive of a poor survival outcome.

In past efforts, we have focussed our efforts on identifying metabolomic changes that accompany a favourable response to systemic therapy [15, 19]. Others have also reported response-related changes in the circulating metabolome [20–23]. There were some limitations to this approach. Most significantly, the absence of a metabolomic response may be insufficient to spur the oncologist to stop treatment. Clinically, this is not unlike what oncologists already encounter when they identify stable disease or even minor progression on follow-up scans; they are often reluctant to stop therapy despite the absence of measurable response because it does not prove therapeutic futility. Here, we describe a biomarker that convincingly demonstrates futility; individuals who repeatedly demonstrate changes in metabolites that accompany tumor progression reliably have short progression-free survivals. Interestingly, the appearance of the biomarker in patients with hepatocellular carcinoma on a different drug was also predictive of a shorter PFS. We have only identified one preclinical report of *in vivo* metabolomic changes associated with tumor progression that were diametrically opposite metabolomic changes corresponding to a treatment response [24].

Bertini et al. reported that baseline metabolomic features in serum predict survival in patients receiving chemotherapy [25]. We were unable to identify such features in our study. Our approach is different, involving identification of changes in the metabolome over time associated with tumor progression. Therefore, rather than identifying a predictive or prognostic biomarker, we have identified a reactive biomarker, its appearance corresponding to tumor growth.

The metabolome is extremely sensitive to changes in physiology and health and, due to the co-relationship of various metabolites, metabolites change in recognizable patterns. This is what makes our approach to treatment monitoring attractive. On the other hand, the metabolome is sensitive to factors such as diet, environment and genetics. The approach we have taken does, to some degree, control for this since the biomarker depends on treatment-related changes from baseline. Ideally, one could follow serial changes in the metabolome indefinitely even in patients who initially responded, for the early identification of chemoresistance. This would perhaps limit cumulative toxicities. However, the biomarker does not appear to work as reliably at time points farther from pre-treatment baseline. More studies on serial blood samples will be required to determine if progression-associated changes in the metabolome can appear during intervals. We were limited to three post-treatment time points in our study; extending the sample collection over a longer period may be helpful in this regard.

Clinically, it is typical for an oncologist to continue therapy in patients with stable disease unless dose-limiting toxicities appear. The problem is that, in these circumstances, it is difficult to determine if the chemotherapy is actually benefiting the patient or whether the disease cadence is just slow. This is particularly problematic when cytostatic drugs are administered (as opposed to cytotoxic drugs). We explored whether the metabolomic biomarker would be able to discriminate individuals who are progressing, but this was not possible. Regardless, patients who did not fulfill metabolomic criteria of progression or response had an intermediate prognosis, which effectively distinguishes them from patients in whom further treatment is futile.

In conclusion, we have described a novel method for identifying patients who will not benefit from palliative chemotherapy. The metabolomic changes that signify tumor progression are apparent within a week of initiating treatment, and decisions to cease treatment based on a confirmatory test can be made within 4 weeks. Further testing will be required to determine how generalizable the biomarker is for other tumor types and other antineoplastic therapies. Ideally, a standardized protocol for blood sampling should be devised. Finally, to bring such a biomarker to clinical fruition, it will be essential to adapt the test to quantify metabolites with minimal measurement variance. While the biomarkers generated in our studies may not be definitive, we have described a discovery and validation workflow that can be utilized for future use.

## MATERIALS AND METHODS

### Patients and samples

This study was approved by the Health Research Ethics Board of Alberta Cancer Committee (HREBA-CC 14-0074). Samples used were taken from patients with metastatic colorectal cancer participating in an international, multicenter, double-blind, randomized controlled phase III study comparing third-line cetuximab and cetuximab plus brivanib (NCIC-CTG CO.20) [16]. Patients in that trial had KRAS wild-type, metastatic CRC previously treated with a fluoropyrimidine, irinotecan and oxaliplatin. Patients also had progression or contradictions to these treatments within 6 months of completing treatment. Patients were randomized into one of two treatment arms: cetuximab + brivanib (*N* = 376) or cetuximab + placebo (*N* = 374). Patient samples were accrued between 2008 and 2011. Plasma samples were collected in K2-EDTA lavender top tubes (Thermo Fisher Scientific, Waltham, Massachusetts) at baseline (prior to treatment initiation), and during the first 12 weeks after treatment (weeks 1, 4 and 12). Patients were not required to fast.

### Chemotherapy response assessment

CT scans were performed at baseline and every 8 weeks after treatment initiation until objective response was documented. Treatment response was evaluated and response was classified according to RECIST 1.0 criteria [7]. According to those criteria, there were no complete responses. In the cetuximab only arm, 7.2% had a partial response, 43.6% had stable disease, and 38% had progression. In the cetuximab + brivanib arm 13.6% had a partial response, 50% had stable disease, and 21.5% had progression. Patients who died without documented objective radiological progression were excluded from this study.

### Study design

This was a nested case-control biomarker discovery design. The discovery cohort consisted of 68 patients who had blood samples from all four time points: baseline, and weeks 1, 4 and 12. Samples were selected from each study arm, with approximately equal representation from each age group, gender and response category. Samples from each response category were matched by gender and age (within 5 years). The validation cohort consisted of 120 randomly selected patients who had blood samples from baseline, and at minimum two other time points. Samples with approximately equal representation from each study arm were selected. Patient characteristics of the discovery and validation groups are summarized in Table 1.

### Gas Chromatography-Mass Spectrometry (GC-MS)

Metabolite extraction and derivatization methods described by Bligh and Dyer were used. Briefly, a two-phase mixture of methanol and chloroform (2:1) was transferred to individual aliquoted sample tubes. Separating into aqueous and organic layers, the aqueous layer tubes were transferred and dried in vacuum (SpeedVac, Eppendorf, Germany) in order to concentrate and shift the solvent towards the gas phase. Sample metabolites were derivatized by adding methoxyamine-hydrochloride in pyridine solution, and N-Methyl-N-(trimethylsilyl) trifluoroacetamide (MSTFA; Millipore-Sigma, Oakville, Canada) a silylating agent to each tube. GC-MS grade hexane was used to dilute the samples, and any solid- or micro-particles were removed by centrifuging the tubes. Spiked-in internal standards consisted of deuterium-labeled metabolites representative of diverse chemical classes with a wide range of retention indices. Deuterated metabolites included phenylalanine D-5, D-glucose-D7, malonic acid-D4, glycine-D5, palmitic acid-D31, L-leucine-D10, L-lysine-D9, and myo-inositol-D6 at concentrations at the mid-range of the linear part of each of their standard curves.

GC-MS was performed on a Bruker Scion 436 GC-MS (Bruker Daltonics INC, Fremont, United States). The MS was operated in the 50–800 m/z range. Mass spectra were processed and quantified using Metabolite Detector software (Version 2.06, Technische Universität Carolo-Wilhelmina zu Braunschweig, Braunschweig, Germany). Serial plasma samples from each patient were deliberately included in the same batch, but randomly distributed. In the discovery experiments, each batch was designed to include approximately equal representation of each treatment arm, sex and age group. Pooled quality controls were distributed throughout each batch, prior to each series of 10 experimental samples. Similarly, a series of aliphatic alkanes were used as calibration standards (n-decane, n-docosane, n-dodecane, n-hexacosane, n-nonadecane, n-pentadecane, n-triacontane (Millipore-Sigma, Oakville, Canada)), and these were distributed throughout each batch prior to the pooled quality control controls.

### Data analysis

Mass spectrometry peaks were analyzed using Metabolite Detector software (Version 2.06, Technische Universität Carolo-Wilhelmina zu Braunschweig, Braunschweig, Germany). Peaks were initially normalized based on spiked-in internal controls. Metabolites were identified based on retention indices, retention times, and individual ions, using the GOLM metabolite database and NIST library as a reference. A second normalization step was performed using median fold-change methods. Missing values were replaced with the minimum quantitative value in the data set. To correct for inter-batch variation in GC-MS, the ComBat approach (through the Bioconductor R package “sva”) in R environment (version 3.3) was applied. ComBat also allows for the removal of batch-dependent noise.

Changes in metabolite concentration from baseline were calculated for each post-treatment time point. Instead of submitting metabolite concentrations to further analysis (as is the usual approach), changes in metabolites were used as the dependent variable. Data were analyzed using SIMCA-P+ software (version 15.0, Umetrics AB, Umeå, Sweden). To identify potential outlier samples and to detect intrinsic data structures, a principal component analysis (PCA) was performed. Subsequent supervised analyses were performed using orthogonal partial least squares discriminant analysis (O-PLS-DA). Metabolites selected were based on variable importance on projection (VIP) thresholds set to maximize R^2^Y and Q^2^Y values and to minimize the difference between them, as previously described [15, 19]. To assess the performance of supervised multivariate models, R^2^Y and Q^2^Y scores were used for measurement of the dataset variance covered by the model, and the predictability of the model in 7-fold cross-validation.

Progression-free survival (PFS) was calculated from the date of treatment initiation until radiographic assessment of disease progression. Overall survival (OS) was calculated from the date of treatment initiation until patient death. Survival curves were estimated using the Kaplan–Meier method, compared using the log-rank test using GraphPad Prism (version 7.0, GraphPad Software Inc, San Diego, United States).

### Pathway analysis

Metabolites that changed with treatment were put into functional context using pathway analysis. Metabolites that differentially changed (from baseline) as a function of treatment response category were submitted to MetaboAnalyst (Version 4.0; https://www.metaboanalyst.ca). MetaboAnalyst allows for the identification of perturbed metabolic pathways from its HMDB-derived archive of over 7000 human metabolite entries and 350 metabolic pathways. Network and pathway analyses were performed in order to generate chemical KEGG identifiers. Subsequently, KEGG pathways were manually examined to extrapolate potential effects of tumor progression on metabolic pathways.

## SUPPLEMENTARY MATERIALS



## Abbreviations

AUROC: area under ROC curve
CRC: colorectal cancer
CT: computed tomography
GC-MS: gas chromatography-mass spectrometry
O-PLS-DA: orthogonal partial least squares discriminate analysis
PCA: principal component analysis
ROC: receiver operating characteristic
