# Supplementary material for: Early detection of treatment futility in patients with metastatic colorectal cancer
Source: Oncotarget. 2022 Jan 7;13:61–72. doi: 10.18632/oncotarget.28165 (PMC8746015; doi:10.18632/oncotarget.28165)
Supplement: Supplementary file 1 [file oncotarget-13-28165-s001.pdf]

Early detection of treatment futility in patients with metastatic colorectal cancer

SUPPLEMENTARY MATERIALS

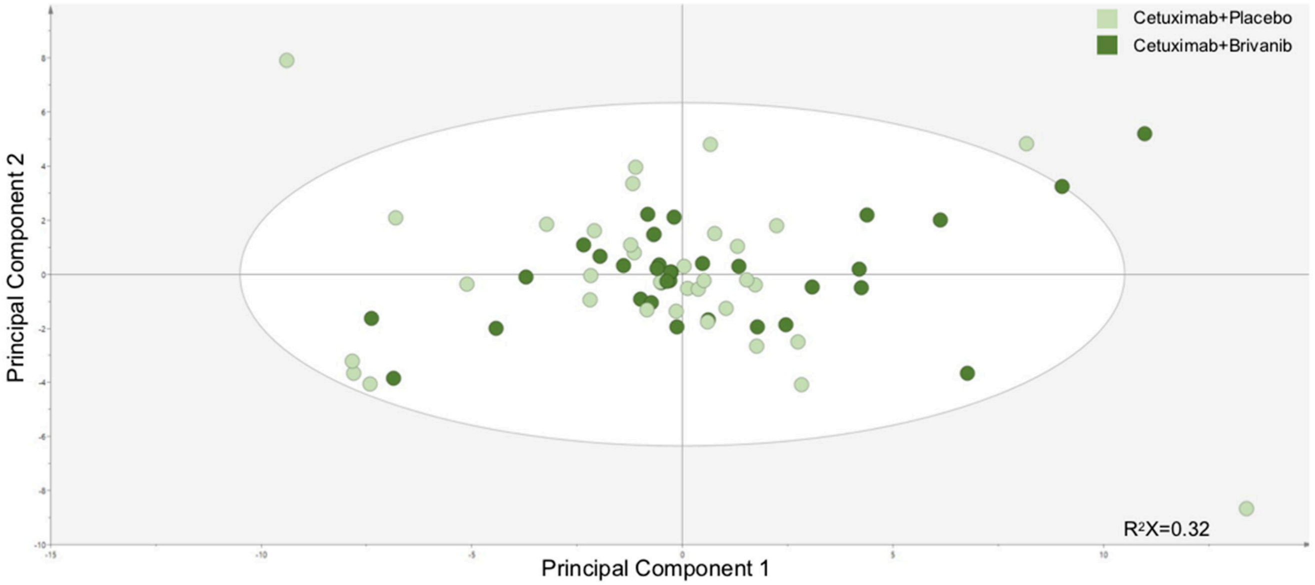

Supplementary Figure 1: PCA scatter plot depicting changes in plasma metabolites as a function of treatment arm.

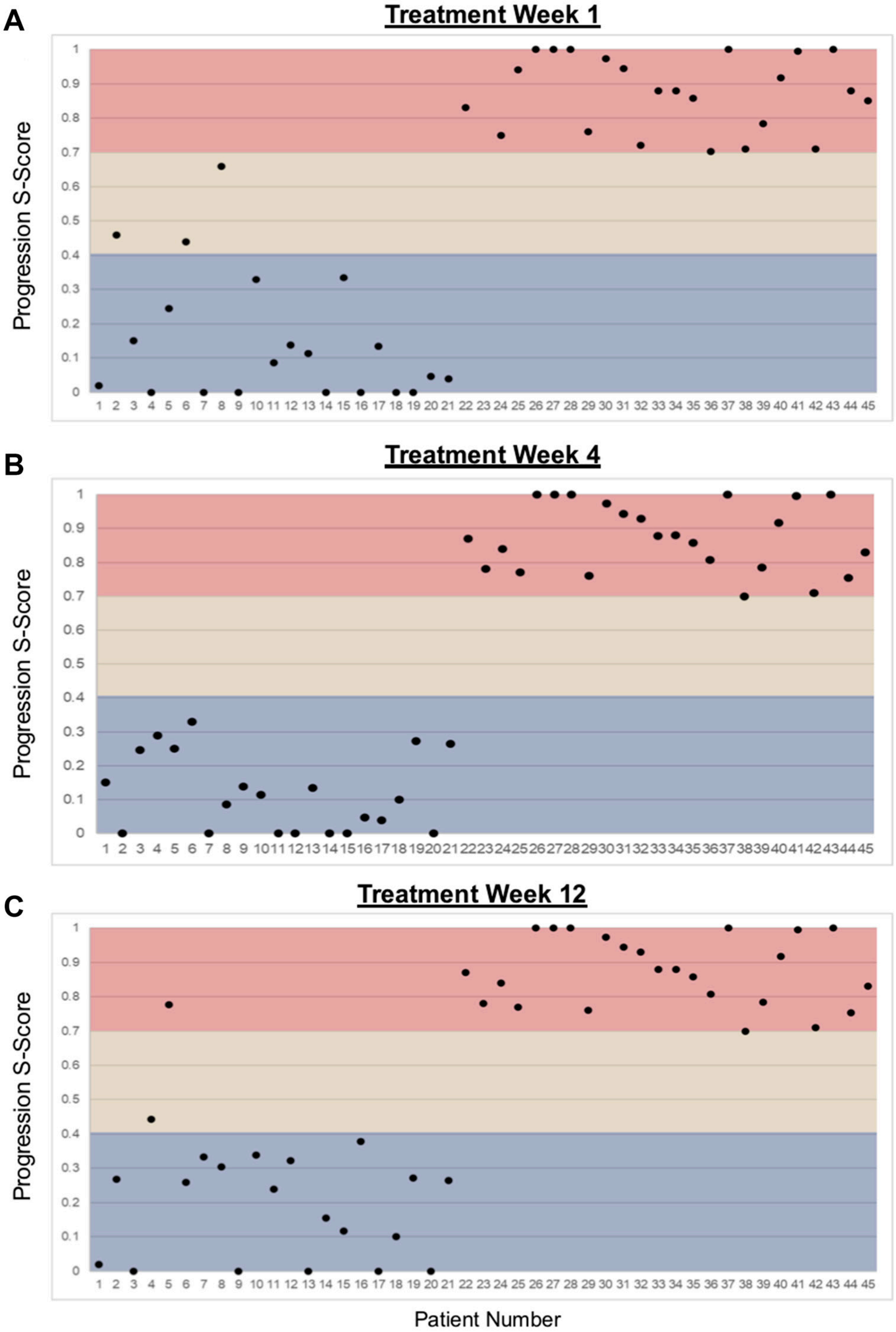

Supplementary Figure 2: S-scores based on the Week 1 model in patients with radiographic PD and PR. (**A**) Week 1 S-scores. (**B**) Week 4 S-scores. (**C**) Week 12 S-scores.
